# Age-related changes in DNA methylation in a sample of elderly Brazilians

**DOI:** 10.1186/s13148-025-01821-3

**Published:** 2025-02-05

**Authors:** Hayley Welsh, Caio M. P. F. Batalha, Weili Li, Nadja C. Souza-Pinto, Yeda A. O. Duarte, Michel S. Naslavsky, Esteban J. Parra

**Affiliations:** 1 Department of Anthropology, University of Toronto at Mississauga, Mississauga, Canada; 2 Department of Biochemistry, University of São Paulo, São Paulo, Brazil; 3 The Centre for Applied Genomics, Hospital for Sick Children, Toronto, Canada; 4 Medical-Surgical Nursing Department, School of Nursing, University of São Paulo, São Paulo, Brazil; 5 Epidemiology Department, Public Health School, University of São Paulo, São Paulo, Brazil; 6 Department of Genetics and Evolutionary Biology, University of São Paulo, São Paulo, Brazil

**Keywords:** DNA methylation, Illumina EPIC array, Molecular aging, Longitudinal study

## Abstract

**Background:**

Age-related changes in DNA methylation (DNAm) play a critical role in regulating gene expression. However, most epigenome-wide association studies have predominantly focused on individuals of European descent. This study aims to characterize longitudinal changes in DNAm patterns in a cohort of elderly Brazilian participants.

**Methods:**

DNAm profiles were collected approximately nine years apart from 23 elderly Brazilian individuals using the Illumina Infinium MethyationEPIC BeadChip. Using mixed-effects models, we examined changes in DNAm patterns using both quantitative age and binary timepoint (e.g., baseline vs. follow-up) as predictors of interest to identify differentially methylated positions (DMPs). Significant DMPs were compared with a list of previously identified age-related DMPs. Differentially methylated regions (DMRs) were also identified using *DMRcate*. Gene ontology (GO) pathway enrichment analyses were performed to explore the functional significance of identified DMPs and DMRs.

**Results:**

Of the 586,229 autosomal probes included in the differential methylation analyses, 2768 significant (FDR < 0.05) age-associated DMPs (aDMPs) and 2757 significant (FDR < 0.05) timepoint-associated DMPs (tpDMPs) were identified. Of the 2768 aDMPs, 1471 were replicated from previous studies. Of the 1297 non-replicated CpGs, 77.4% were exclusive to the EPIC array. The DMR analyses identified 305 age-associated DMRs (aDMRs) and 372 timepoint-associated DMRs (tpDMRs). Both aDMPs and aDMRs exhibited age-related hypermethylation within CpG islands and promoter regions of the genome, whereas hypomethylation predominantly occurred in interCGI and intergenic regions and introns. The GO enrichment analyses identified several neurological and cognition-related pathways enriched for hypermethylated CpG islands, many of which were mapped near transcription start sites and first exon regions.

**Conclusions:**

This longitudinal study identified age-associated and timepoint-associated DMPs and DMRs in a sample of elderly Brazilians. Most of the non-replicated CpGs were found to be on the new EPIC array, suggesting that more age-related studies using the EPIC array are required to validate these CpGs. The GO pathway enrichment analyses identified age-related enrichment of several gene sets related to cognitive and physical decline in elderly populations. The enrichment of these sites could provide evidence for age-related neurodegeneration and cognitive decline in elderly populations.

**Supplementary Information:**

The online version contains supplementary material available at 10.1186/s13148-025-01821-3.

## Introduction

The process of aging can be understood as the progressive deterioration of biological system integrity arising from the accumulation of cellular level changes [1]. Variability in human aging and onset of age-related disease is not only based on genetics, but also epigenetics. DNA methylation (DNAm) is an important epigenetic mechanism that plays a role in gene expression and regulation [2, 3]. Methylation occurs when the enzyme DNA methyltransferase covalently bonds a methyl group (CH_3_) to the fifth carbon ring of a cytosine within a cytosine-guanine dinucleotide (CpG site), creating 5-methylcytocine (5mC) during synthesis [3–6]. Within the human genome there are approximately 28 million CpG sites, of which 60–90% are typically methylated [3, 5]. However, while most CpG sites are heavily methylated, CpG islands—areas of the genome containing higher densities of CpG sites—are typically resistant to methylation [2, 3, 5]. Dysregulation in DNAm is associated with premature aging and a variety of age-related illnesses including cancer, diabetes, cardiovascular disease, metabolic disease, and neurological diseases [5, 7]. Therefore, analyzing DNAm can provide important information regarding disease risk and health outcomes.

When it comes to age-related DNAm changes, studies have found a general pattern for DNAm over the lifespan, with methylation levels increasing in blood and saliva during the first year of life [8–11]. The DNAm changes observed in the first year of life primarily occur at intragenic regions, CpG island shores, enhancers, and promoters that lack CpG islands [9, 12]. Throughout the developmental period of childhood and adolescence, DNAm levels increase rapidly [13–15]. DNAm changes then stabilize in early to middle adulthood [15]. From mid-adulthood to advancing age, overall DNAm levels decrease and interindividual variability increases [16]. This phenomenon is commonly referred to as “epigenetic drift,” characterized by non-directional changes in DNAm over time that involve both hypomethylation and hypermethylation events, resulting from both genetic and environmental factors [17, 18].

Age-associated hypomethylation, or demethylation, typically occurs in heterochromatic regions of the genome, such as repetitive elements and transposons, which contain the majority of CpG sites in the human genome [19]. Additionally, region- and site-specific hypermethylation has also been broadly observed in the aging genome [20]. Typically, age-related hypermethylation occurs at CpG islands, and more particularly CpG island promoters [21, 22]. Hypermethylation of CpG island promoters has been associated with the silencing of genes and suppression of transcription [21, 22]. Age-related changes in DNAm can be assessed through examining differentially methylated positions (DMPs) and differentially methylated regions (DMRs) [23]. Biological relevance can be assigned to DMPs and DMRs using gene enrichment analysis [23].

When considering age-related changes in DNAm, comparatively few longitudinal studies have evaluated how DNAm profiles change over time in elderly populations [18] and ethnically diverse populations [24, 25]. In this study, we examined age-related changes in genome-wide DNAm in a sample of elderly Brazilian participants from the Health, Well-being and Aging (Saúde, Bem-estar e Envelhecimento; SABE) study cohort [26, 27]. Two samples taken ~ 9 years apart were available for each individual. Differential methylation analysis was performed using mixed-effects models with (1) quantitative age or (2) binary timepoint as predictors of interest. Positions and regions identified as being differentially methylated were then used for gene pathway enrichment analysis to identify differentially methylated pathways.

## Materials and methods

### Study participants and samples

The current study used whole blood samples obtained from the SABE study cohort, which is comprised of census-withdrawn elderly individuals from São Paulo, Brazil. This study cohort has been followed up every five years since 2000, with DNA first collected in 2010, and has been previously described in detail in recent genomic studies [27, 28]. Samples from 24 elderly adults (13 males and 11 females) were taken at two timepoints (9 ± 0.71 years apart) for a total of 48 samples. The first timepoint is from the 2010 collection wave, performed from June 2010 through September 2012, and the samples for the second timepoint were collected during the COVID-19 monitoring project, performed from June 2020 through April 2021. The 24 individuals were 67.41 ± 5.52 years of age (mean ± standard deviation) at timepoint one, and 76.41 ± 6.17 at timepoint two. Global ancestry analyses in the SABE cohort have indicated evidence of genetic similarities with European, West African, Indigenous American, and East Asian populations, as well as broad variation in individual ancestral proportions, in agreement with the known demographic history of the Brazilian population [27].

### Blood collection and processing

Genomic DNA was extracted from whole peripheral blood samples collected in EDTA tubes. DNA extraction and purification followed manufacturer’s recommended protocols, using the Qiagen AutoPure LS kit with Gentra automated extraction for samples collected at the first timepoint and manual extraction for samples collected at the second timepoint. The change in extraction techniques was necessitated by the discontinuation of the equipment, however, the same commercial reagents were used for both timepoints. DNA was quantified with a Nanodrop spectrometer and diluted to 50 ng/uL. Whole-genome sequencing (WGS) data for the samples described above are also available.

### Characterization of DNA methylation using the EPIC array

Approximately 1000 ng of human genomic DNA was used for bisulfite conversion. In August of 2021, DNA methylation was measured using the Infinium MethylationEPICv1.0 array at The Centre for Applied Genomics (TCAG, Hospital for Sick Children, Toronto, Ontario, Canada), in accordance with the protocols recommended by Illumina (San Diego, California, USA).

### Processing of DNA methylation DNA

Using the R/Bioconductor packages *Meffil* (v1.1.0), *RnBeads* (v2.6.0), *minfi* (v1.34.0), and *wateRmelon* (v1.32.0), the methylation data were imported and processed, and quality control (QC) analyses were performed. First, we used *Meffil* to infer sex, which was then compared to reported sex. Using the 59 SNP probes that are available as part of the EPIC array, the concordance between the methylation intensities of the samples and the corresponding genotype calls extracted from their WGS data was calculated. Then, using the *RnBeads* QC pipeline, we performed comprehensive sample-level and probe-level QC. Specifically, probes were removed if their target sequences overlap with a SNP at any base, if they are known to be cross-reactive, and if more than 5% of the samples had a missing value. Additionally, the iterative Greedycut algorithm was used to filter out samples and probes using a detection p-value threshold of 0.01. We used the *wateRmelon* package to extract bead numbers from the IDAT files and calculated the proportion of samples with a bead number < 3. Probes were removed if more than 5% of samples had a low bead number (< 3). We also computed detection *p* values using out-of-band probes empirical distribution with the pOOBAH() function in the *SeSAMe* (v1.14.2) R package, with a *p* value threshold of 0.05, and the combine.neg parameter set to TRUE. The pOOBAH was done in parallel with the previously mentioned QC steps, and the resulting probes flagged in both analyses were combined and removed from the data. These QC steps were previously described in Welsh et al. [29].

One sample was determined to be low quality based on QC, and due to the paired study design, we also excluded the second sample from the same individual. Therefore, a total of *n* = 46 samples from *n* = 23 individuals (13 males and 10 females) were included in the differential methylation analyses. Post QC, a total of 666,485 probes remained, and we further excluded 15,119 sex chromosome probes. Since probes with low variation across all samples are less likely to show significant association, and to reduce the burden of multiple-testing correction [30], we decided to further exclude 65,137 probes for which the standard deviations of the beta values were in the lowest 10% of distribution. In summary, a total of 586,229 probes were analyzed.

### Differential cell composition estimation

Different cell types may exhibit different methylation patterns, and differential cell type proportions across study samples can lead to spurious associations [31]. The proportions of cell types in each of the study samples were estimated using the method proposed by Salas et al. [32], as implemented in the R package *FlowSorted.Blood.EPIC*. Proportions of the 6 cell types, CD8 + T cell (CD8T), CD4 + T cell (CD4T), natural killer (NK), B cell (Bcell), monocytes (Mono) and neutrophils (Neu) were estimated for all 46 samples.

### Normalization and creation of cell composition adjusted M-values

Post QC, we first performed data normalization using *SeSAMe* [33] as suggested in Welsh et al. [29]. We then computed adjusted M-values by removing the effect of differential cell type compositions through a regression-based approach, as described in Jones et al. [34]. Briefly, (1) *SeSAMe* normalized beta values were regressed on the estimated cell proportions for CD8T, CD4T, NK, Bcell, and Mono; estimated proportions for Neu were not included as a predictor due to multicollinearity, (2) the residuals from the linear regression models were extracted and the mean beta values were added to the residuals to obtain adjusted beta values, (3) the adjusted beta values were then converted to adjusted M-values. The adjusted M-values reflect sample methylation levels after removing the effect of differential cell type proportions.

### Differential methylation analyses

Mixed-effects models were used for the differential methylation analysis. Two models were evaluated using the adjusted M-values modeled as the response variable, the first using quantitative age as the predictor of interest and the second using timepoint as a binary predictor of interest. For both mixed-effects models, a linear model was fitted with individual ID as a random effect using the dream() function in the R/Bioconductor package *variancePartition* (v1.33.11) [35], to account for the correlation between the two measurements from the same individual. Additionally, variables related to technical variation such as array slide (Sentrix_ID) and position (Sentrix_Position) were also fitted as random effects. Since including individual ID as a random effect can account for subject-invariant variables such as sex and ancestry, they were not included as covariates. Similarly, smoking status was also found to be invariant between the two timepoints and was likewise not included as a covariate. Therefore, the final model for quantitative age was Adjusted M-values ~ Age + (1|Sentrix_ID) + (1|Sentrix_Position) + (1|Individual ID) and the final model for timepoint was Adjusted M-values ~ Binary Timepoint + (1|Sentrix_ID) + (1|Sentrix_Position) + (1|Individual ID). To account for multiple hypothesis testing, the Benjamini-Hochberg FDR corrected p-values were calculated, and probes with FDR-adjusted *p*-value < 0.05 were considered statistically significant at the epigenome-wide level.

DMR analyses for each model were performed using the R package *DMRcate* (v2.16.1) [36]. Using the rmSNPandCH() function in *DMRcate*, an additional *n* = 5172 probes were removed, leaving 581,057 probes for the DMR analyses. Because *DMRcate* uses *limma* for linear modeling, we altered the cpg.annotate() function code to replace the *limma* lmFit() function with the *variancePartition* dream() function. The CpGannotate object generated from the altered code was then processed using the dmrcate() function. A minimum number of three CpGs per region was used.

Genomic annotation was performed for both DMPs and DMRs using the R/Bioconductor package *annotatr* (v1.28.0). *Annotatr* provided CpG annotations (i.e., CpG island, shore, shelf, and interCGI), genic annotations (i.e., promoter, exon, intron, 1to5kb, 5UTR, 3UTR and intergenic), and gene annotations [37]. Shores are defined as 2 Kb upstream/downstream from the ends of the CpG island, shelves are defined as 2 Kb upstream/downstream from the farthest upstream/downstream limits of CpG shores, and interCGI regions, also known as open sea regions [37], are defined as the remaining genomic regions (i.e., > 4 Kb upstream/downstream from the ends of a CpG island). Some CpGs have multiple neighboring genes, meaning a single CpG might receive multiple genic annotations [38]. To mitigate this potential bias in the graphical representations of the data, fractional counting was used, where each genic annotation was divided by the total number of annotations for a given DMP or DMR (i.e., if a DMP or DMR mapped to a promoter region of gene A and gene B, each annotation was counted as 0.5) [38]. Chi-squared tests were then employed using the chisq.test() function, to determine if the observed CpG annotation distributions significantly (*p* < 0.05) differ from expected values for both DMPs and DMRs.

### Gene expression/enrichment

Gene set enrichment analyses were conducted in order to identify potential pathways enriched in differentially methylated probes/regions. Using the R/Bioconductor package *missMethyl* (v1.36.0) gene ontology (GO) pathway enrichment analyses were performed. This package maps CpGs to genes and tests for gene enrichment using the Wallenius’ non-central hypergeometric test, which accounts for gene-length bias [23, 39]. For each model (i.e., quantitative age and timepoint), gene enrichment analyses were performed using both the gometh() and goregion() functions which mapped the DMPs and DMRs to a list of 22,593 a priori genes, respectively. Enrichment analyses were performed on the total number of significant DMPs and DMRs as well as for all hyper- and hypomethylated DMPs and DMRs, hypermethylated DMPs and DMRs annotated to CpG islands, hypomethylated DMPs and DMRs annotated to interCGI (open sea) regions, and hypermethylated DMPs and DMRs annotated to CpG islands and mapped near transcription start sites (TSS200 and TSS1500) and 1st exon regions. Therefore, a total of 24 enrichment analyses were performed. All GO enrichment analyses were run using the default options of ‘prior.prob = TRUE’ and ‘fract.counts = TRUE’ to account for two additional sources of bias in the pathway analysis: (1) the differing number of probes per gene, and (2) CpGs could be annotated to multiple genes. The pathways for gometh() and goregion() include biological process (BP), cellular component (CC), and molecular function (MF) [39]. Pathways with an FDR-adjusted *p*-value < 0.05 were considered statistically significant.

### Replication of previous studies

We compiled lists of significant age-associated DMPs (aDMPs) reported in previously published studies. The lists considered include the aDMPs of 33 studies collected by, and available through, the open platform EWAS Atlas [40] as well as lists from 15 additional studies [13, 41–54]. The EWAS Atlas did not include the exhaustive lists of aDMPs for six of the 33 studies; therefore, the full lists of these six of the studies were also considered [55–60]. These studies were conducted on a variety of tissue types, populations, age ranges, Illumina array types (i.e., 27 k, 450 k, and EPIC array), and study designs (e.g., cross-sectional, longitudinal, twin-study design). We compared these lists with our significant age- and timepoint-associated DMPs in order to determine the proportion of replicated CpGs.

## Results

In this study, the genome-wide methylation profiles of *n* = 46 whole blood samples, from *n* = 23 Brazilian participants, were examined. Two DNAm profiles collected ~ 9 years apart from each individual were obtained using the Illumina Infinium MethyationEPIC BeadChip. After data preprocessing and *SeSAMe* normalization, 586,229 autosomal probes were eligible for analysis. Using the methylation status of these probes, we analyzed the effects of quantitative age and timepoint on DNAm profiles.

### Longitudinal EWAS on age

#### Differential methylation

Using a mixed-effects model with quantitative age as the predictor of interest, a total of 2768 age-associated significantly (FDR < 0.05) differentially methylated positions (aDMPs) were identified. Using the more stringent Bonferroni correction (*p* < 0.05), only *n* = 22 significant probes were identified. The aDMPs identified account for 0.47% of investigated probes. The list of significant aDMPs can be found in File S1. We sought to determine the extent to which the significant aDMPs identified in the current study are replicated from previous research that considered age-related changes in DNAm. Comparing the significant aDMPs with age-associated probes identified by previous studies, 1471/2768 (53.1%) were replicated. Of the 1297 non-replicated CpGs, 1004 (77.4%) were exclusive to the EPIC array. The list of non-replicated aDMPs can be found in File S2.

Significant aDMPs with a logFC > 0 were considered hypermethylated (increasing with age) and significant aDMPs with a logFC < 0 were considered hypomethylated (decreasing with age). Nearly an equal number of hyper- and hypomethylated aDMPs were identified, with *n* = 1381 hypermethylated probes and *n* = 1387 hypomethylated probes. Differentially methylated regions (DMRs) were identified using *DMRcate* [36]. After filtering out an additional *n* = 5172 probes using the rmSNPandCH() function, as recommended by *DMRcate*, a total of 581,057 EPIC array v1.0 autosomal probes were considered for the DMR analysis. *DMRcate* identified 2843 significant (FDR < 0.05) aDMPs. Using the 2843 significant probes, 305 age-associated DMRs (aDMRs) were identified. Of the 305 aDMRs, 271 were found to be hypermethylated (mean difference > 0) and 32 were found to be hypomethylated (mean difference < 0). The list of aDMRs can be found in File S3.

**Fig. 1:**
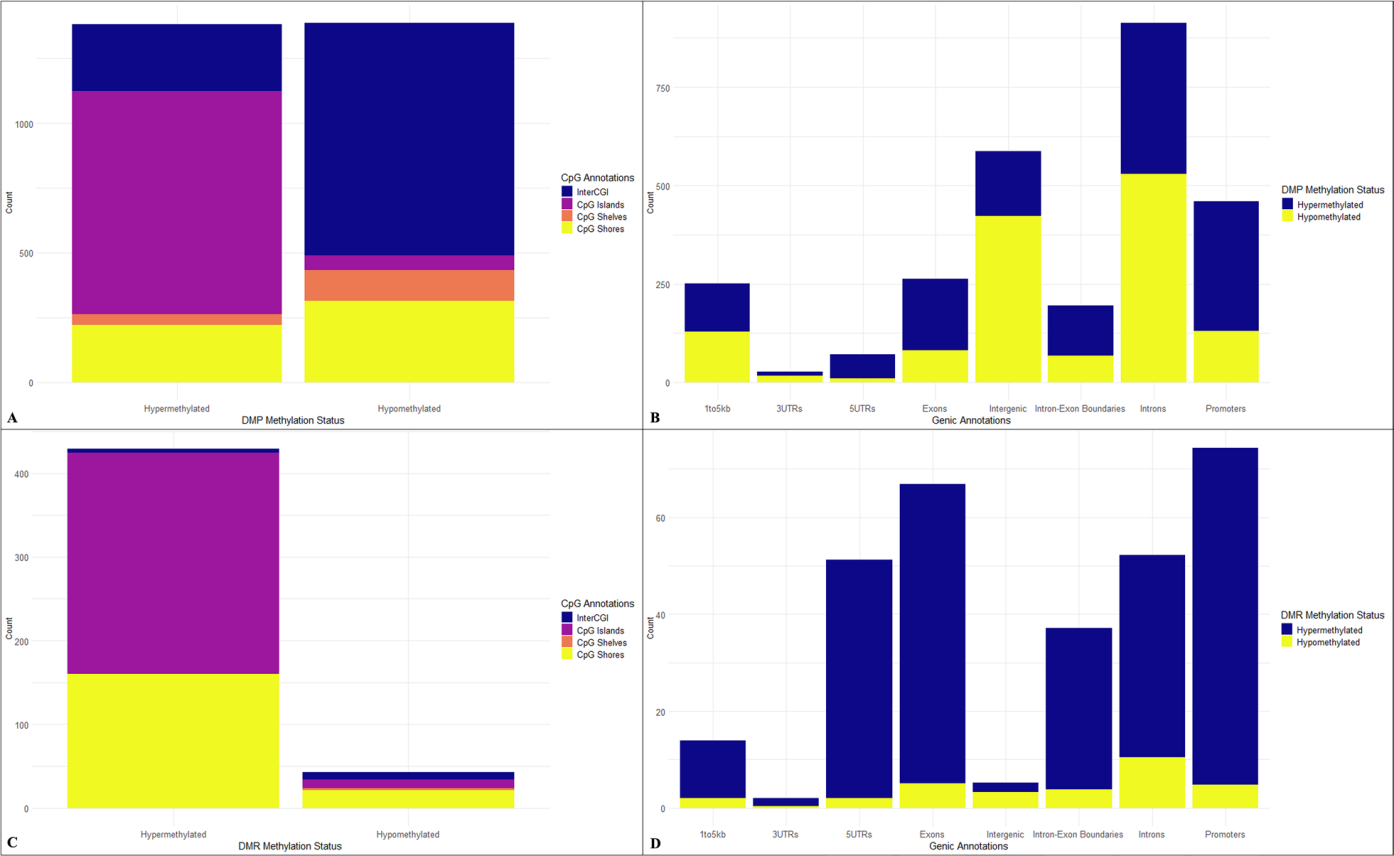
Distributions of CpG and genic annotations for significant aDMPs and aDMRs. **A** Distribution of CpG annotations for significant aDMPs, **B** Distribution of genic annotations for significant aDMPs using fractional counting, **C** Distribution of CpG annotations for significant aDMRs, **D** Distribution of genic annotations for significant aDMRs using fractional counting

Using CpG and genic annotations, the genomic region distributions of the hyper- and hypomethylated aDMPs and aDMRs were analyzed. Figure 1A and Table 1 show that hypermethylated aDMPs are primarily located within CpG islands, while hypomethylated aDMPs are primarily located within interCGI (open sea) regions. The chi-square results in Table 1 also show that more CpG island shelves and shores are hypomethylated than expected. Similarly, Fig. 1B and Table 2 show that there are proportionally more hypermethylated aDMPs in promoter, 5′UTR, exon, and intron–exon boundary regions, while hypomethylated aDMPs are primarily found in introns and intergenic regions. The aDMR results follow the same trend as the aDMP results, with hypermethylated aDMRs being primarily annotated to CpG islands, and hypomethylated aDMRs being primarily annotated to interCGI regions (Fig. 1C; Table 3). The aDMR chi-square results in Table 3 similarly show that along with interCGI regions, there are more hypomethylated CpG island shelves and shores than expected. When considering genic annotations, there are more hypermethylated aDMRs than hypomethylated aDMRs in genic regions, but the opposite is observed in intergenic regions (Fig. 1D). With respect to the genic regions, when the hyper- and hypomethylated aDMRs are plotted separately (Fig. S1), it becomes clear that, compared to hypomethylated aDMRs, the relative proportions of hypermethylated aDMRs are higher within promoters, exons, and 5′UTR regions, than within intergenic regions. This result is further supported by the chi-square test results in Table 4 (using fractional counting) and Table S1 (without fractional counting/raw counts).

**Table 1:** Chi-Square results for CpG annotations using significant aDMPs

	Hypermethylation (Observed)	Hypermethylation (Expected)*	Hypomethylation (Observed)	Hypomethylation (Expected)*
InterCpG	257	576	898	579
Islands	861	457	55	459
Shelves	43	81	119	81
Shores	220	267	315	268
Total	1381		1387	

*Rounded to the nearest whole number

X-squared = 1117.5, df = 3, *p*-value < 2.2e−16

**Table 2:** Chi-Square results for genic annotations using significant aDMPs (fractional counting used)

	Hypermethylation (Observed)*	Hypermethylation (Expected)*	Hypomethylation (Observed)*	Hypomethylation (Expected)*
1to5kb	122	125	129	126
3′UTR	10	13	17	14
5′UTR	61	35	9	35
Exon	183	132	81	132
Intergenic	165	293	422	294
Intronexonboundaries	128	98	68	98
Intron	383	456	530	457
Promoter	329	230	131	230
Total	1381		1387	

*Rounded to the nearest whole number

X-squared = 319.82, df = 7, *p*-value < 2.2e-16

**Table 3:** Chi-Square results for CpG annotations using aDMRs

	Hypermethylation (Observed)	Hypermethylation (Expected)*	Hypomethylation (Observed)	Hypomethylation (Expected)*
InterCpG	5	13	9	1
Islands	264	249	10	25
Shelves	0	3	3	0
Shores	160	164	21	16
Total	429		43	

*Rounded to the nearest whole number

X-squared = 92.62, df = 3, *p*-value < 2.2e-16

**Table 4:** Chi-Square results for genic annotations using aDMRs (fractional counting used)

	Hypermethylation (Observed)*	Hypermethylation (Expected)*	Hypomethylation (Observed)*	Hypomethylation (Expected)*
1to5kb	12	13	2	1
3′UTR	2	2	0	0
5′UTR	49	46	2	5
Exon	62	60	5	7
Intergenic	2	4	3	1
Intronexonboundaries	33	33	4	4
Intron	42	47	10	5
Promoter	69	66	5	8
Total	271		31	

*Rounded to the nearest whole number

X-squared = 22.239, df = 7, *p*-value = 0.00231

#### Gene enrichment analyses

To further investigate the functional significance of the aDMPs and aDMRs identified in this study, we performed Gene ontology (GO) enrichment pathway analyses. Gene enrichment analysis of aDMPs and aDMRs was conducted using 22,593 GO terms in the *missMethyl* package. Using all 2768 aDMPs, gometh() identified *n* = 61 significantly (FDR < 0.05) enriched GO terms: 42 biological process (BP), 13 cellular component (CC), and six molecular function (MF). Using the topGSA() function, cell–cell signaling (GO:0007267), nervous system development (GO:0007399), and neurogenesis (GO:0022008) were identified as the top three most significant terms (Fig. S2A). When restricting the input to significant hypermethylated aDMPs, *n* = 55 significantly (FDR < 0.05) enriched GO terms were identified: 33 BP, four CC, and 18 MF. The top three pathways for this analysis related to DNA-binding and RNA polymerase (GO:0000981, GO:0003700, and GO:0000977) (Fig. S2B). Using only hypermethylated aDMPs annotated to CpG islands, *n* = 101 significantly (FDR < 0.05) enriched GO terms were identified: 55 BP, 18 CC, and 28 MF. The same top three pathways were identified as in the previous analysis (Fig. S2C), likely because, as shown in Table 1, the majority (62.3%) of age-associated hypermethylated CpGs are annotated to CpG islands. Restricting the gometh() analysis to hypermethylated aDMPs annotated to CpG islands and found near TSSs (i.e., TSS200 & TSS1500) and the 1st exon (i.e., promoter region), yielded *n* = 51 significant GO terms (Fig. S2D). No enriched GO terms were identified when using all hypomethylated aDMPs. However, two significantly (FDR < 0.05) enriched co-occurring GO terms were identified when using hypomethylated open sea (interCGI) aDMPs: extracellular matrix (GO:0031012) and collagen-containing extracellular matrix (GO:0062023). All significant gometh() results can be found in File S4.

Using all *n* = 305 aDMRs, a total of *n* = 22 significantly (FDR < 0.05) enriched GO terms were identified: 17 BP, four CC, and one MF (Fig. S3A). When only using the hypermethylated aDMRs, *n* = 36 significant (FDR < 0.05) GO terms were identified: 25 BP, six CC, and five MF (Fig. S3B). Similarly, when only considering hypermethylated aDMRs annotated to CpG islands, *n* = 33 significant (FDR < 0.05) GO terms were identified: 23 BP, five CC, and five MF (Fig. S3C). These three analyses identified the same top four pathways: nervous system development, neurogenesis, generation of neurons (GO:0048699), and synaptic membrane (GO:0097060). Restricting the goregion() analysis to hypermethylated aDMRs annotated to CpG islands and found near TSSs (TSS200 and TSS1500) and 1st exon, yielded *n* = 22 significant GO terms (Fig. S3D). No pathways were identified when using hypomethylated aDMRs. All significant goregion() results can be found in File S5.

### Longitudinal EWAS on binary timepoint

#### Differential methylation

Using a mixed-effects model with binary timepoint as the predictor of interest, a total of 2757 timepoint-associated significantly (FDR < 0.05) differentially methylated positions (tpDMPs) were identified. Using the more stringent Bonferroni correction (*p* < 0.05), only *n* = 9 significant probes were identified. The tpDMPs identified account for 0.47% of investigated probes. The list of significant tpDMPs can be found in File S1. While using age and timepoint as a predictor of interest do not necessarily address the same questions, few EWAS looking at changes in DNAm over time have used timepoint as a binary predictor [61, 62]. Therefore, as with the aDMPs, we sought to determine how many of the significant tpDMPs identified in the current study are replicated from previous research that considered age-related changes in DNAm. Comparing the significant probes identified with age-associated probes identified by previous studies, 807/2757 (29.3%) of the tpDMPs were replicated. Of the 1950 non-replicated CpGs identified, 1334 (68.4%) were exclusive to the EPIC array. The list of non-replicated tpDMPs can be found in File S2.

Of the 2757 significant tpDMPs identified, *n* = 778 were found to be hypermethylated (logFC > 0) and *n* = 1979 were found to be hypomethylated (logFC < 0). *DMRcate* identified 2820 significant (FDR < 0.05) tpDMPs. Using these significant probes, 372 timepoint-associated DMRs (tpDMRs) were identified, of which 60 were found to be hypermethylated (mean difference > 0) and 312 were found to be hypomethylated (mean difference < 0). The list of DMRs can be found in File S3.

Using CpG and genic annotations, the genomic region distributions of the hyper- and hypomethylated tpDMPs and tpDMRs were analyzed. In concordance with the aDMP results, Fig. S4A and Table S2 show that there are more hypomethylated tpDMPs than expected within interCGI (open sea) regions, whereas there are more hypermethylated tpDMPs than expected in CpG islands, and these differences are significant based on the chi-square test. When considering tpDMP genic annotations, all genomic categories are disproportionately more hypomethylated (Fig. S4B), but similarly to the analysis based on age, the number of hypomethylated probes in intergenic regions is higher than expected (Table S3). Though, unlike with the aDMP analysis, there is no disproportionately higher-than-expected hypermethylation occurring in promoter regions (Table S3). The tpDMR results for CpG annotations are also in line with the aDMR results, with more hypermethylated tpDMRs mapped to CpG islands and more hypomethylated tpDMRs mapped to interCGI regions than expected, based on the chi-square test results (Fig. S4C; Table S4). However, in contrast to the aDMR results, there are slightly more hypermethylated tpDMR in shores than expected (Table S4). As with the tpDMP genic annotations, there are more hypomethylated tpDMRs than hypermethylated tpDMRs for all the genomic categories, but similar to the analyses focused on age, the frequency of hypermethylated tpDMRs is higher in promoters, exons, and 1to5kb regions compared to intergenic regions (Fig. S5B). Additionally, the chi-square results show that there are more hypermethylated tpDMRs annotated to promoter, 1to5kb, and 5′UTR regions than expected (Tables S5 & S6). Though it should be noted, that only the tpDMR chi-square results using the raw DMR genic annotation counts (Table S6), rather than fractional genic annotations counts (Table S5), are statistically significant.

#### Gene enrichment analyses

To further investigate the functional significance of the tpDMPs and tpDMRs identified in this study, we performed gene ontology (GO) enrichment pathway analyses. None of the gometh() analyses using the tpDMPs yielded significantly enriched pathways. When considering tpDMRs, no pathways were identified when considering all *n* = 372 tpDMRs or the *n* = 60 hypermethylated tpDMRs and *n* = 312 hypomethylated tpDMRs separately. However, *n* = 39 significant (FDR < 0.05) GO terms were identified when restricting the analysis to hypermethylated tpDMRs annotated to CpG islands: 28 BP and 11 MF. The majority of the pathways were related to DNA-binding, transcription and RNA regulation, with the top three pathways being DNA-templated transcription (GO:0006351), regulation of DNA-templated transcription (GO:0006355), and RNA biosynthetic process (GO:0032774) (Fig. S6A). Restricting the goregion() analysis to hypermethylated tpDMPs annotated to CpG islands and found near TSSs (TSS200 and TSS1500) and 1st exon, yielded *n* = 9 significant GO terms (Fig. S6B). These terms included the previously mentioned top three pathways, as well as RNA metabolic process (GO:0016070), organic cyclic compound metabolic process (GO:1901360), organic cyclic compound biosynthetic process (GO:1901362), regulation of RNA metabolic process (GO:0051252), transcription regulator activity (GO: 0140110), and regulation of RNA biosynthetic process (GO:2001141). No pathways were identified when using hypomethylated tpDMRs mapped to interCGI regions. All significant goregion() results can be found in File S5.

### Overlap between aDMPs and tpDMPs

**Fig. 2:**
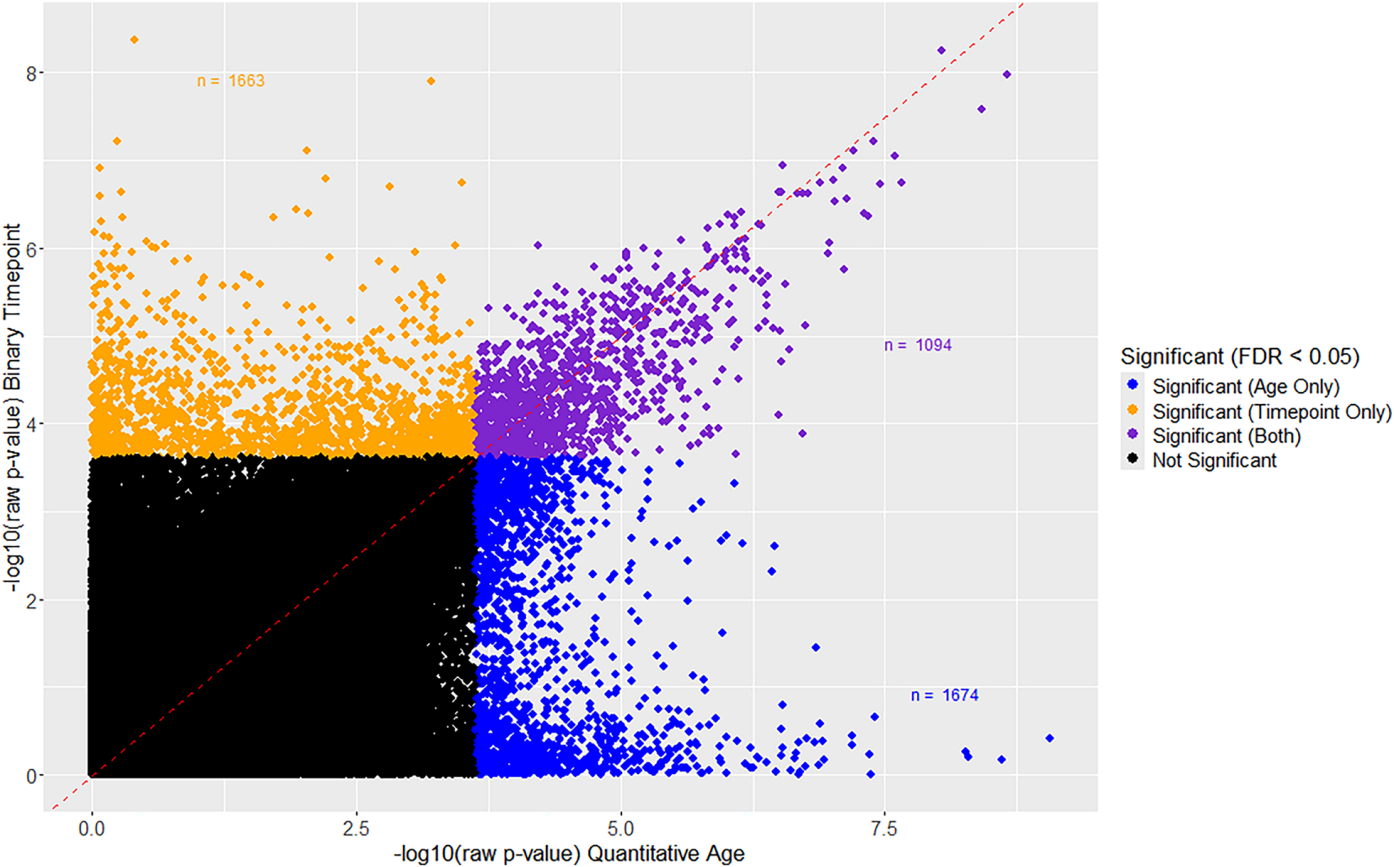
Compares raw (unadjusted) p-values between aDMPs and tpDMPs. DMPs significant after FDR adjusted are highlighted (FDR < 0.05). Orange represents significant DMPs only identified using timepoint as the predictor, blue represents significant DMPs only identified using quantitative age as the predictor, and purple represents the DMPs that overlap within both models

When looking at the overlap of DMPs between the two models (i.e., aDMPs and tpDMPs), *n* = 1094 significant DMPs were identified (Fig. 2). All of the overlapping DMPs were found to be directionally concordant (i.e., hypermethylated or hypomethylated), with *n* = 262 hypermethylated probes and *n* = 832 hypomethylated probes. When considering the significant non-overlapping probes of each model, the majority (> 90%) of probes were directionally concordant between the two models.

## Discussion

In this study, we explored longitudinal changes in DNAm patterns in a sample of elderly Brazilians. We considered both age- and timepoint-associated changes in DNAm. Overall, the results for both models are concordant in indicating a trend toward increased methylation with age within CpG islands, and decreased methylation within interCGI regions. Similarly, we observed increased methylation in promoters, exons and 5′UTR regions, when compared to introns and intergenic regions. The model based on timepoint tends to identify more hypomethylated probes and regions than the model based on age, but there is a substantial overlap between the probes identified in both models. When performing gene enrichment analyses with DMPs, only age-associated DMPs (aDMPs) provided significant results. Given that most comparable EWAS have considered quantitative age as opposed to binary timepoint, we will be primarily discussing the results of the age-associated analyses. Through this discussion we will explore the results of the age-associated analyses and contextualize them within the current research regarding age-related changes in DNAm.

Of the *n* = 2768 aDMPs identified, 53.1% were replicated from previous studies. *N* = 551 were replicated from the open platform EWAS Atlas [40] and an additional *n* = 920 were replicated from other previous studies (File S2) [13, 41–60]. Of the 1297 non-replicated aDMPs, 1004 (77.4%) are available on the EPIC array, but not in Illumina’s previous methylation arrays. The lack of replication for these CpGs likely largely results from the fact that majority of previous studies use the older generation Illumina arrays (i.e., 27 k array and/or 450 k array), as only 7/33 studies included in the EWAS Atlas and only five out of the additional 15 study datasets considered probes available on the EPIC array. Additional factors that could have affected replication include differences in age ranges, study design (i.e., cross-sectional vs. longitudinal), tissue type, sample size, covariates considered, and population [63]. Aside from differences in arrays, age ranges are a particularly important factor to consider since changes in DNAm have been shown to follow different patterns throughout the life course [15]. Additionally, EWAS have predominantly been performed using populations of European descent [24, 25]. Therefore, additional longitudinal studies using the EPIC array and ancestrally diverse populations may be required to validate the remaining 1297 non-replicated age-associated probes identified in this study.

### DNA methylation patterns

The association between aging and changes in DNAm has been studied for decades [20]. It was originally proposed that a spontaneous loss of 5-methylcytosines (5mCs) with age led to abnormal expression of genes in cells/tissues that are normally repressed [64]. Instead, a gradual loss of 5mC has been reported, with 5mC content being highest in early life and then decreasing gradually with age [65]. When considering changes that occur from mid-adulthood to advancing age, studies have shown overall DNAm levels decrease and interindividual variability increases [15, 16]. Additionally, region- and site-specific hypermethylation, typically occurring at CpG islands, has also been broadly observed in the aging genome [20]. As expected, the patterns of age-associated changes observed in the current study broadly follow the patterns of epigenetic drift typical for individuals of advancing age (elderly individuals). Hypermethylated aDMPs and aDMRs were found to be enriched in CpG islands and promoter regions (See Fig. 1). In contrast, clear patterns of hypomethylation were observed for interCGI (open sea) and intergenic regions.

### Enrichment analyses

Many pathways were enriched for hypermethylated probes and regions, especially those mapped to CpG islands. Several of the significantly enriched GO terms identified in this study have also been identified by other studies looking at age-associated changes in DNAm. For example, cell–cell signaling [53, 56, 66, 67], nervous system development [53, 55, 68], and neurogenesis [56] have been widely identified in other aging studies. Marttila et al. [50] also reported many of the same transcription and RNA regulatory GO terms identified in the current study.

Along with neurogenesis and generation of neurons, many other neurological/cognitive related GO terms were identified in the current study, such as neuron differentiation (GO:0030182), central nervous system (GO:0007417), brain development (GO:0007420), learning or memory (GO:0007611), and memory (GO:0007613). All of these processes are critically involved in aging and neurodegeneration [69] and have been identified in DNAm studies examining differential methylation patterns related to Alzheimer’s disease [70]. Additionally, the majority of the hypermethylated aDMPs and aDMRs that were annotated to CpG islands were also mapped near TSSs (i.e., TSS200 and TSS1500) and first exon regions (See File S3 & S4). Increased methylation near TSSs and first exon regions is often associated with transcriptional silencing [71] and gene expression suppression [20, 72]. Therefore, these GO terms may provide evidence for the accumulation of epigenetic changes relating to cognitive decline in elderly individuals.

When considering hypomethylated positions and regions, only age-related hypomethylated interCGI probes identified enriched GO pathways. These pathways were related to extracellular matrix and collagen functioning. Enrichment in extracellular matrices has been found to be associated with aging and a variety of age-related diseases, such as Pulmonary fibrosis [73], osteoarthritis [74, 75], and intervertebral disk degeneration [76]. Therefore, the results of this study suggest that the accumulation of age-related changes in DNAm, which primarily involved hypermethylation at CpG islands and hypomethylation at interCGI regions, may be affecting the regulation of gene expression in important regulatory regions of the genome.

## Conclusion

In conclusion, this study explored age-associated changes in DNA methylation in a sample of elderly Brazilians. The inclusion of longitudinal data and the focus on a population underrepresented in biomedical studies are two of the main strengths of the study. The major limitation is the small sample size, but despite this limitation, the majority of our age-associated probes overlap with the findings of previous EWAS of age. Most of the non-replicated CpGs were found to be on the new EPIC array, suggesting that more age-related studies using the EPIC array are required to validate these CpGs. Using gene ontology pathway enrichment analyses, we identified age-related enrichment of several gene sets, many of which are related to developmental and transcriptional regulation. The enrichment of these sites could be directly associated with age-related cognitive and physical decline in elderly populations, though larger longitudinal studies are required to properly investigate the relationship between these epigenetic changes and gene expression.

## Supplementary Information

Additional file1.

Additional file2.

Additional file3.

Additional file4.

Additional file5.

supplementary Figures.

supplementary Tables.

## Data Availability

Due to privacy concerns, individual-level data cannot be made publicly available. However, data requests, describing the proposed use(s) of the data, can be sent to Hayley Welsh, the corresponding author for the study, or Dr. Esteban Parra (esteban.parra@utoronto.ca).

## Abbreviations

aDMP: Age-associated differentially methylated position
aDMR: Age-associated differentially methylated region
BP: Biological process
CC: Cellular component
CpG: Cytosine-guanine dinucleotide
DMP: Differentially methylated position
DMR: Differentially methylated region
DNAm: DNA methylation
EWAS: Epigenome-wide association study
FDR: False discovery rate
GO: Gene ontology
interCGI: Areas of the genome > 4 Kb from a CpG island
Kb: Kilobases
logFC: Log fold change
MF: Molecular function
QC: Quality control
TCGA: The Centre for Applied Genomics
SABE: Saúde, Bem-estar e Envelhecimento
tpDMP: Timepoint-associated differentially methylated position
tpDMR: Timepoint-associated differentially methylated region
TSS: Transcription start site
WGS: Whole-genome sequencing
1–5 kb: 1–5 Kilobases
3′UTR: 3′ Untranslated region
5mC: 5-Methylcytocine
5′UTR: 5′ Untranslated region
