# Supplementary material for: Age-related changes in DNA methylation in a sample of elderly Brazilians
Source: Clin Epigenetics. 2025 Feb 5;17:17. doi: 10.1186/s13148-025-01821-3 (PMC11796210; doi:10.1186/s13148-025-01821-3)
Supplement: Supplementary file 6 — supplementary Figures. [file 13148_2025_1821_MOESM6_ESM.docx]

**Age-related changes in DNA methylation in a sample of elderly Brazilians**

**Hayley Welsh^1*^, Caio M. P. F. Batalha^2^, Weili Li^3^, Nadja C. Souza-Pinto^2^, Yeda A. O. Duarte^4,5^, Michel S. Naslavsky^6^, Esteban J. Parra^1^**

^1^ *Department of Anthropology, University of Toronto at Mississauga, Mississauga, Canada*

^2^ *Department of Biochemistry, University of São Paulo, São Paulo, Brazil*

*^3^ The Centre for Applied Genomics, Hospital for Sick Children, Toronto, Canada*

*^4^ Medical-Surgical Nursing Department, School of Nursing, University of São Paulo, São Paulo, Brazil
^5^ Epidemiology Department, Public Health School, University of São Paulo, São Paulo, Brazil*

*^6^ Department of Genetics and Evolutionary Biology, University of São Paulo, São Paulo, Brazil ^*^Corresponding author: hayley.welsh@mail.utoronto.ca*

**Supplementary Figures S1-S6**


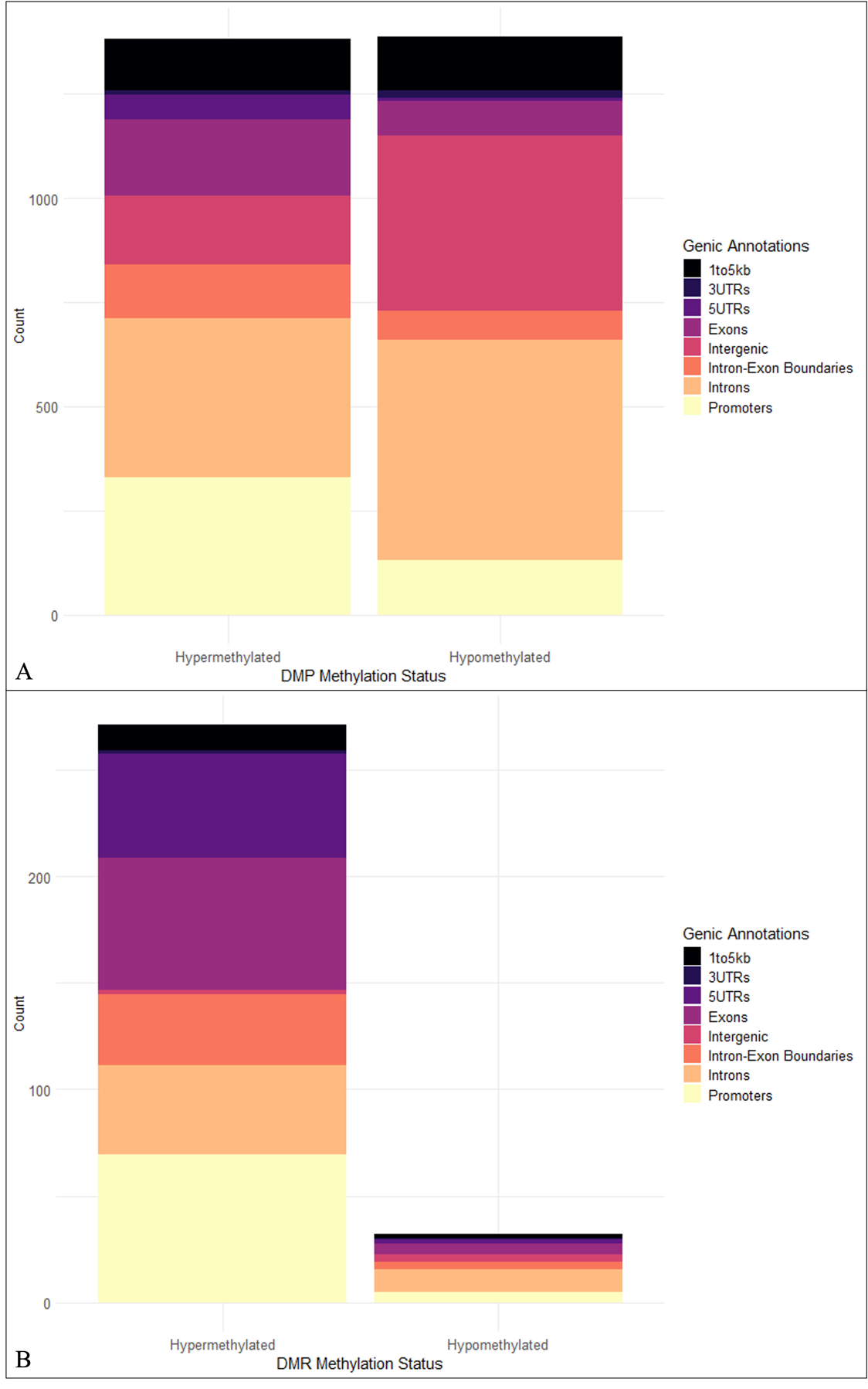


**Fig. S1. Distribution of genic annotations for significant aDMPs and aDMRs**. A) Distribution of genic annotations for significant aDMPs B) Distribution of genic annotations for significant aDMRs.


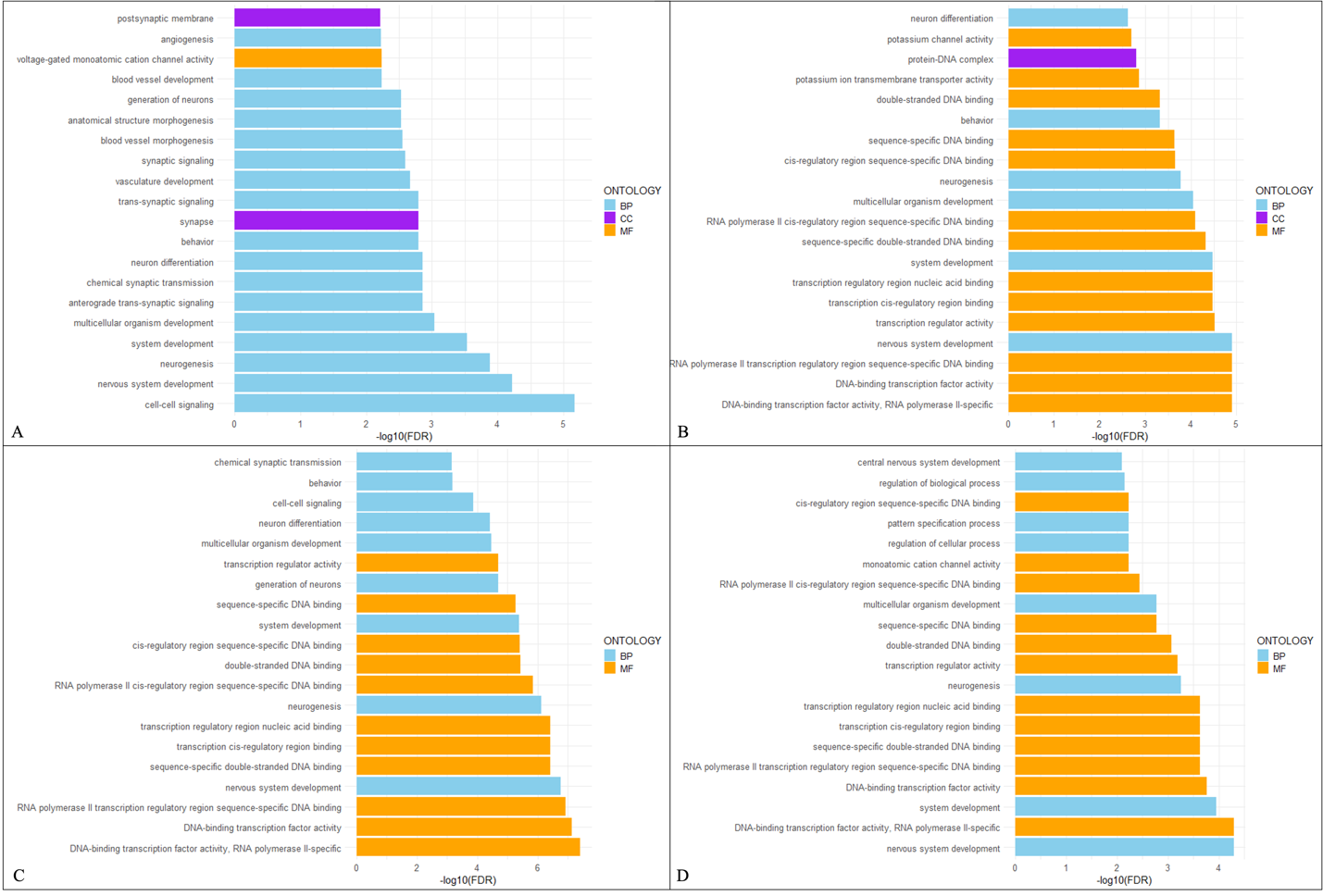


**Fig. S2. Displays top 20 most significantly enriched pathways from gene ontology enrichment (GO) analyses using gometh().** A) GO results using all aDMPs B) GO results using all hypermethylated aDMPs C) GO results using all hypermethylated aDMPs annotated to CpG islands D) GO results using all aDMPs annotated CpG islands and mapped to transcription starts sites (TSS200 and TSS1500) and 1^st^ exon regions. BP = biological process; CC = cellular component; MF = molecular function.


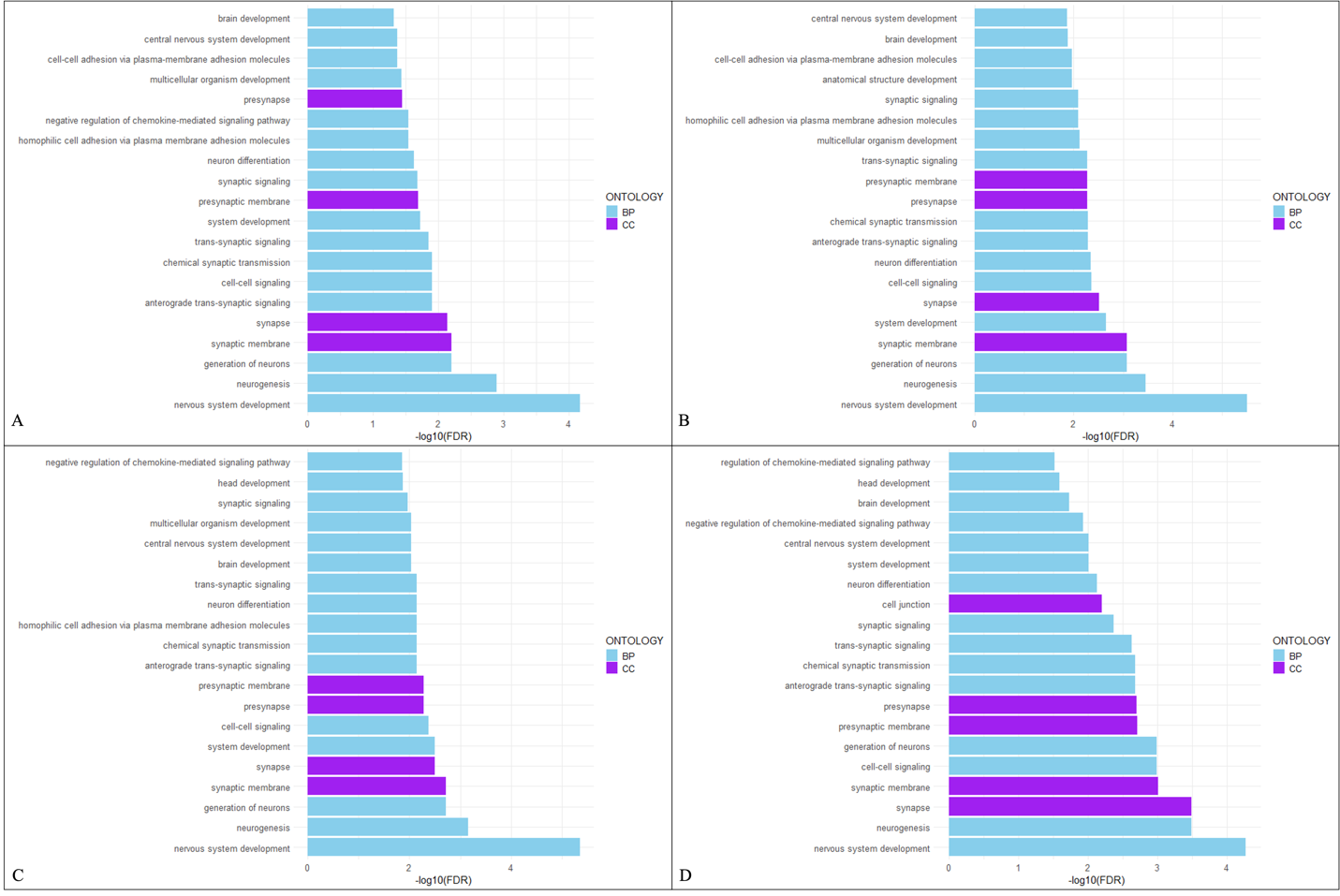


**Fig. S3. Displays top 20 most significantly enriched pathways from gene ontology enrichment (GO) analyses using goregion().** A) GO results using all aDMRs B) GO results using all hypermethylated aDMRs C) GO results using all hypermethylated aDMRs annotated to CpG islands D) GO results using all aDMRs annotated to CpG islands and mapped to transcription starts sites (TSS200 and TSS1500) and 1^st^ exon regions. BP = biological process; CC = cellular component.


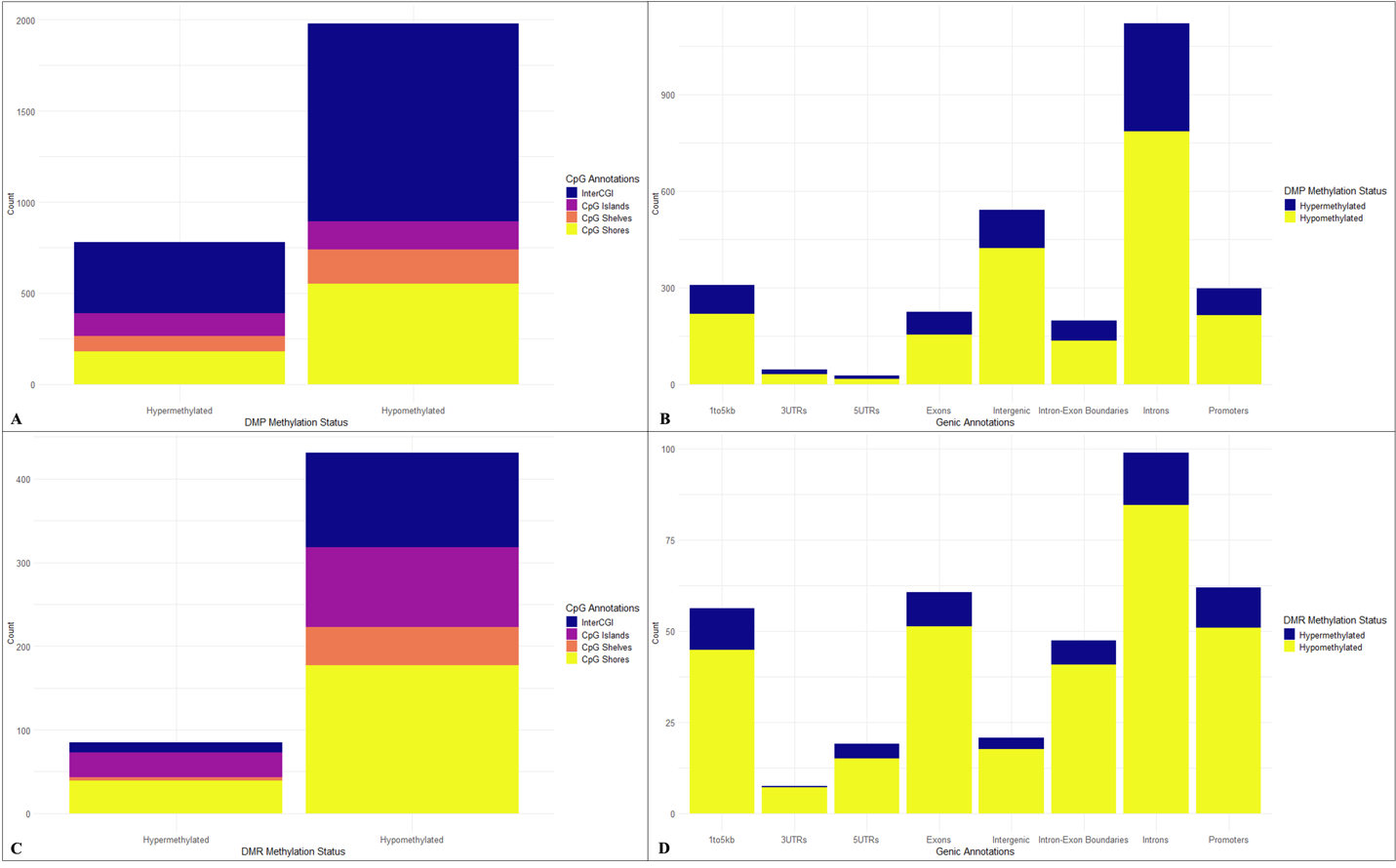


**Fig. S4. Distributions of CpG and genic annotations for significant tpDMPs and tpDMRs.** A) Distribution of CpG annotations for significant tpDMPs B) Distribution of genic annotations for significant tpDMPs C) Distribution of CpG annotations for significant tpDMRs D) Distribution of genic annotations for significant tpDMRs.


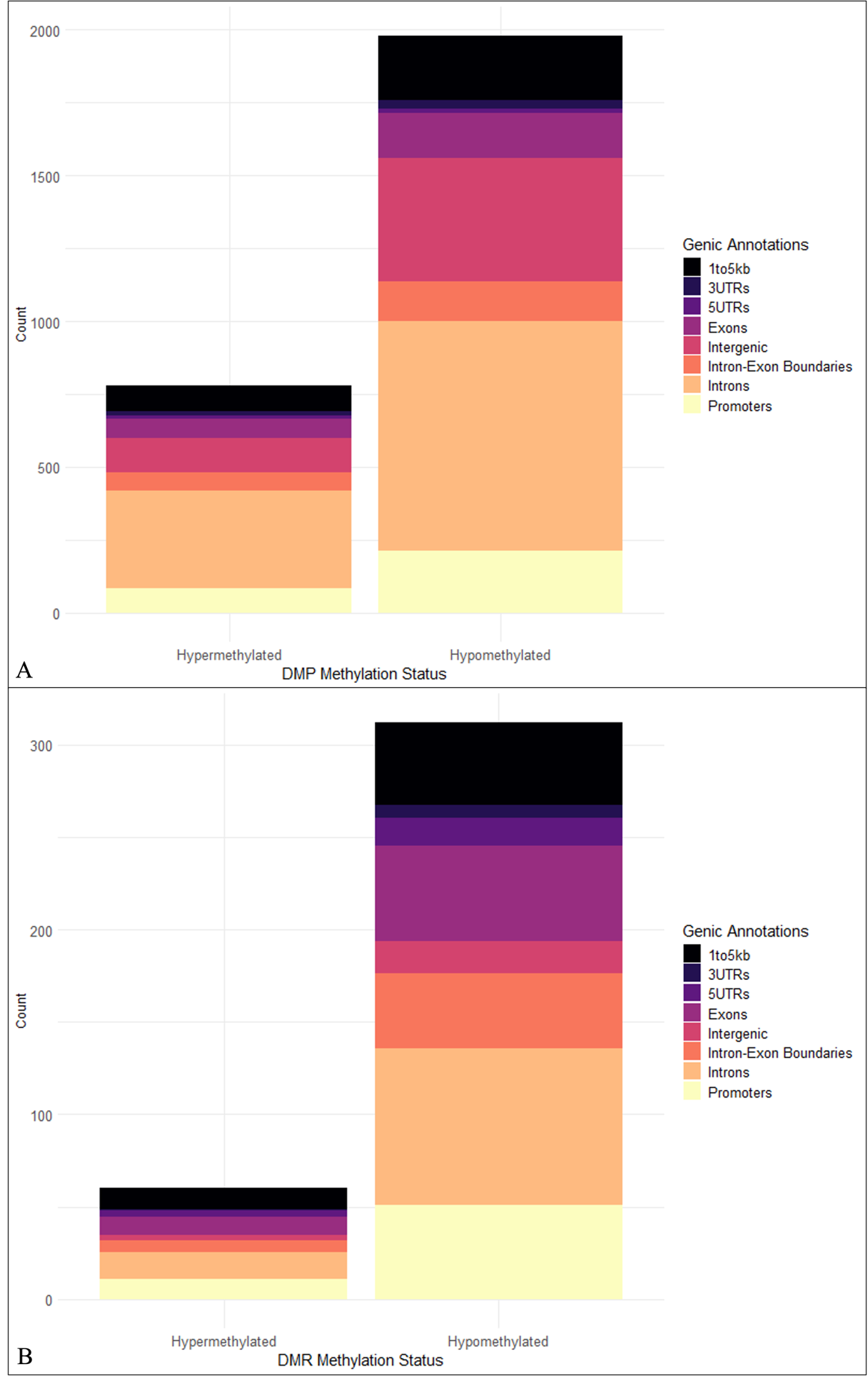


**Fig. S5.** **Distribution of genic annotations for significant tpDMPs and tpDMRs.** A) Distribution of genic annotations for significant tpDMPs B) Distribution of genic annotations for significant tpDMRs.


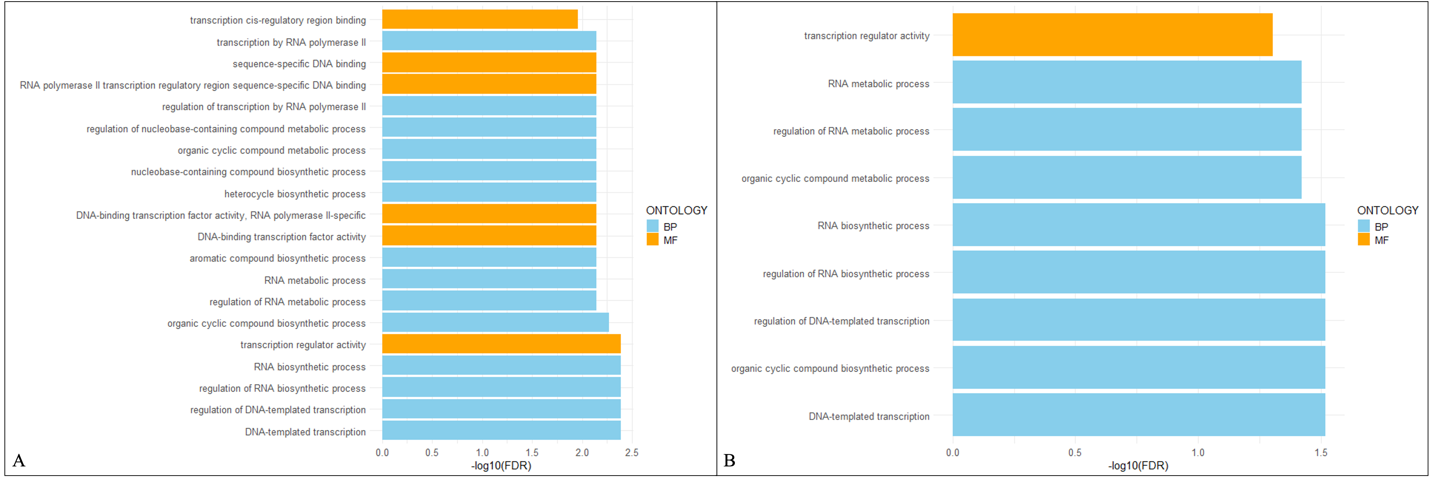


**Fig. S6.** **Displays top 20 most significantly enriched pathways from gene ontology enrichment (GO) analyses using goregion().** A) GO results using all hypermethylated tpDMRs annotated to CpG islands B) GO results using all tpDMRs annotated to CpG islands and mapped to transcription starts sites (TSS200 and TSS1500) and 1^st^ exon regions. BP = biological process; MF = molecular function.
