# Supplementary material for: Age-related changes in DNA methylation in a sample of elderly Brazilians
Source: Clin Epigenetics. 2025 Feb 5;17:17. doi: 10.1186/s13148-025-01821-3 (PMC11796210; doi:10.1186/s13148-025-01821-3)
Supplement: Supplementary file 7 — supplementary Tables. [file 13148_2025_1821_MOESM7_ESM.docx]

**Age-related changes in DNA methylation in a sample of elderly Brazilians**

**Hayley Welsh^1*^, Caio M. P. F. Batalha^2^, Weili Li^3^, Nadja C. Souza-Pinto^2^, Yeda A. O. Duarte^4,5^, Michel S. Naslavsky^6^, Esteban J. Parra^1^**

^1^ *Department of Anthropology, University of Toronto at Mississauga, Mississauga, Canada*

^2^ *Department of Biochemistry, University of São Paulo, São Paulo, Brazil*

*^3^ The Centre for Applied Genomics, Hospital for Sick Children, Toronto, Canada*

*^4^ Medical-Surgical Nursing Department, School of Nursing, University of São Paulo, São Paulo, Brazil
^5^ Epidemiology Department, Public Health School, University of São Paulo, São Paulo, Brazil*

*^6^ Department of Genetics and Evolutionary Biology, University of São Paulo, São Paulo, Brazil ^*^Corresponding author: hayley.welsh@mail.utoronto.ca*

**Supplementary Tables S1-S6**

Table S1. Chi-Square results for genic annotations using aDMRs (raw counts)

|  | Hypermethylation (Observed) | Hypermethylation (Expected)^*^ | Hypomethylation (Observed) | Hypomethylation (Expected)^*^ |
| --- | --- | --- | --- | --- |
| 1to5kb | 112 | 115 | 12 | 9 |
| 3’UTR | 25 | 26 | 3 | 3 |
| 5’UTR | 537 | 516 | 18 | 39 |
| Exon | 676 | 674 | 49 | 51 |
| Intergenic | 2 | 6 | 4 | 0 |
| Intronexonboundaries | 406 | 416 | 37 | 31 |
| Intron | 515 | 540 | 65 | 40 |
| Promoter | 620 | 604 | 29 | 45 |
| Total | 2893 |  | 271 |  |

*Rounded to the nearest whole number.

** X-squared = 70.476, df = 7, p-value = 1.184e-12

Table S2. Chi-Square results for CpG annotations using significant tpDMPs

|  | Hypermethylation (Observed) | Hypermethylation (Expected)^*^ | Hypomethylation (Observed) | Hypomethylation (Expected)^*^ |
| --- | --- | --- | --- | --- |
| InterCpG | 388 | 416 | 1085 | 1057 |
| Islands | 125 | 79 | 156 | 202 |
| Shelves | 84 | 76 | 186 | 194 |
| Shores | 181 | 207 | 552 | 526 |
| Total | 778 |  | 1979 |  |

*Rounded to the nearest whole number.

** X-squared = 44.879, df = 3, p-value = 9.818e-10

Table S3. Chi-Square results for genic annotations using significant tpDMPs (fractional counting used)

|  | Hypermethylation (Observed)* | Hypermethylation (Expected)^*^ | Hypomethylation (Observed)* | Hypomethylation (Expected)^*^ |
| --- | --- | --- | --- | --- |
| 1to5kb | 88 | 87 | 219 | 220 |
| 3’UTR | 13 | 12 | 31 | 32 |
| 5’UTR | 10 | 7 | 16 | 19 |
| Exon | 69 | 63 | 154 | 160 |
| Intergenic | 117 | 153 | 424 | 388 |
| Intronexonboundaries | 63 | 56 | 135 | 142 |
| Intron | 335 | 316 | 786 | 805 |
| Promoter | 83 | 84 | 214 | 213 |
| Total | 778 |  | 1979 |  |

*Rounded to the nearest whole number.

**X-squared = 16.65, df = 7, p-value = 0.0198

Table S4. Chi-Square results for CpG annotations using tpDMRs

|  | Hypermethylation (Observed) | Hypermethylation (Expected)^*^ | Hypomethylation (Observed) | Hypomethylation (Expected)^*^ |
| --- | --- | --- | --- | --- |
| InterCpG | 12 | 21 | 113 | 104 |
| Islands | 30 | 21 | 95 | 104 |
| Shelves | 4 | 8 | 46 | 42 |
| Shores | 39 | 36 | 177 | 180 |
| Total | 85 |  | 431 |  |

*Rounded to the nearest whole number.

** X-squared = 12.441, df = 3, p-value = 0.006017

Table S5. Chi-Square results for genic annotations using tpDMRs (fractional counting used)

|  | Hypermethylation (Observed)* | Hypermethylation (Expected)^*^ | Hypomethylation (Observed)* | Hypomethylation (Expected)^*^ |
| --- | --- | --- | --- | --- |
| 1to5kb | 11 | 9 | 45 | 47 |
| 3’UTR | 1 | 1 | 7 | 7 |
| 5’UTR | 4 | 3 | 15 | 16 |
| Exon | 9 | 10 | 51 | 50 |
| Intergenic | 3 | 3 | 18 | 18 |
| Intronexonboundaries | 7 | 8 | 41 | 40 |
| Intron | 14 | 16 | 84 | 82 |
| Promoter | 11 | 10 | 51 | 52 |
| Total | 60 |  | 312 |  |

*Rounded to the nearest whole number.

** X-squared = 1.4889, df = 7, p-value = 0.9827

Table S6. Chi-Square results for genic annotations using tpDMRs (raw counts)

|  | Hypermethylation (Observed) | Hypermethylation (Expected)^*^ | Hypomethylation (Observed) | Hypomethylation (Expected)^*^ |
| --- | --- | --- | --- | --- |
| 1to5kb | 72 | 52 | 392 | 412 |
| 3’UTR | 7 | 10 | 79 | 76 |
| 5’UTR | 48 | 28 | 203 | 223 |
| Exon | 94 | 112 | 915 | 897 |
| Intergenic | 3 | 3 | 21 | 21 |
| Intronexonboundaries | 83 | 105 | 865 | 843 |
| Intron | 97 | 129 | 1065 | 1033 |
| Promoter | 86 | 52 | 381 | 415 |
| Total | 490 |  | 3921 |  |

*Rounded to the nearest whole number.

** X-squared = 69.096, df = 7, p-value = 2.25e-12
